# Aryl Hydrocarbon Receptor Is Dispensable for the Early Transcriptional Response of Mouse Macrophages to Type I Interferon

**DOI:** 10.1096/fj.202604796R

**Published:** 2026-11-11

**Authors:** Darawan Tabtim‐On, Elodie Segura

**Affiliations:** ^1^ Institut Curie PSL University, INSERM, U932, Immunity and Cancer Paris France; ^2^ Institut Necker Enfants Malades Université Paris Cité, INSERM, U1151 Paris France

## Abstract

Type I interferon (IFN‐I) signaling is essential in the homeostasis and activation of immune cells, and macrophages play a central role in the IFN‐I system. Dysregulation leads to tissue damage and autoimmunity, and IFN‐I signaling needs to be tightly fine‐tuned. One reported mechanism involves interactions with the transcription factor aryl hydrocarbon receptor (AhR). However, it remains to be determined whether AhR is required for the early transcriptional response of macrophages to IFN‐I. Here we used macrophages from genetically deficient mice to investigate the involvement of AhR in the induction of early IFN‐I‐driven responses. We show that constitutive AhR deficiency does not impair the canonical transcriptional response to IFN‐I in bone marrow‐derived macrophages nor the expression of IFN‐stimulated molecules by peritoneal macrophages upon in vivo exposure. These findings contrast with observations made in other cell types, in particular astrocytes and lung cancer cells, and support the notion of a context‐dependent crosstalk between AhR and IFN‐I‐dependent signaling.

## 1 Introduction

Type I interferon (IFN‐I) signaling is essential in the homeostasis and activation of immune cells [1, 2]. Macrophages are immune sentinels playing a central role in this system as primary responders to IFN‐I and crucial regulators of IFN‐I responses [3, 4, 5, 6]. IFN‐I signaling is fine‐tuned through multiple mechanisms, and its dysregulation leads to tissue damage and autoimmunity [7, 8, 9, 10]. One such mechanism involves interactions with the aryl hydrocarbon receptor (AhR), but accumulating evidence indicates that AhR activity is highly context‐dependent with cell type‐specific effects [11, 12]. In particular, whether AhR is involved in the response of macrophages to IFN‐I remains unknown.

Canonical signaling of the IFN‐I receptor (IFNAR) induces the phosphorylation of STAT1 and STAT2 through the activity of JAK1 and TYK2 kinases [13]. STAT1/STAT2 dimers associate with IRF9 to form the ISGF3 complex, resulting in the transcription of a battery of interferon‐stimulated genes [13]. Such genes include, in particular, components of several negative feedback loops [14].

AhR is a ligand‐activated transcription factor containing a basic helix–loop–helix (bHLH) and a Per–Arnt–Sim (PAS) domain [11]. Physiological AhR agonists include a broad variety of indoles and tryptophan catabolites from endogenous, dietary, or commensal microbiota origin [11]. AhR was reported to modulate JAK/STAT signaling downstream of interferon receptors. In mouse and human astrocytes and lung cancer cells, AhR sustains STAT1 signaling driven by IFN‐I or IFN‐γ [15, 16, 17, 18]. In addition, AhR was shown to participate in the negative regulation of IFN‐I signaling in mouse embryonic fibroblasts and macrophages following viral infection, by dampening IFN‐I responses through the induction of the molecule Tiparp [19]. However, whether AhR is required in macrophages for the induction of the early transcriptional response to IFN‐I remains unclear.

## 2 Results and Discussion

To address the role of AhR in interferon‐stimulated transcriptional responses, we used genetically‐deficient macrophages from CD64*AhR knock‐out (KO) mice obtained by crossing AhR^fl/fl^ with CD64‐iCre mice, and macrophages from wild‐type (WT) littermates (AhR^fl/fl^ mice). We have previously shown that AhR is efficiently deleted in peritoneal macrophages in this strain but not in monocytes [20]. Therefore, the genetic construction does not interfere with the initial stages of monocyte‐to‐macrophage differentiation that are dependent on AhR [21].

IFN‐I responses are characterized in macrophages by the rapid induction of interferon‐stimulated genes (ISG) with a majority of genes peaking in the first hours (< 4 h) [22, 23, 24]. To capture early ISG expression with a high signal‐to‐noise ratio for genome‐wide analysis, we exposed bone marrow‐derived macrophages to IFN‐I for 3 h before bulk RNA‐sequencing analysis. In these in vitro conditions, AhR signaling is constitutively activated by traces of tryptophan‐derived AhR agonists from the culture medium [25], modeling the physiological state of tonic AhR activation in vivo. In addition, using a similar in vitro system, we have previously evidenced that AhR deficiency abolishes the early expression (3 h) of a module of IL‐4‐induced genes in monocytes by interfering with the phosphorylation of STAT6 [26].

To assess whether AhR deficiency affects IFN‐I‐driven transcriptional responses in an unbiased manner, we identified differentially expressed genes using a genotype‐by‐treatment interaction test, then performed K‐means clustering. We identified 3 main clusters of genes with similar expression patterns (Figure 1A). The complete list of genes in each cluster is shown in Table S1. Cluster 1 comprised genes overexpressed in WT macrophages compared to AhR‐deficient macrophages. Such genes included known AhR target genes such as *Cyp1b1* and *Mgst2*. Cluster 2 comprised genes down‐regulated upon IFN‐I exposure, independently of the genotype. Pathway enrichment analysis showed enrichment for genes involved in mRNA transcription and metabolic processes (Figure 1B), consistent with the reported repression upon IFN‐I stimulation of transcriptional programs related to metabolic enzymes and macromolecular biosynthesis [27, 28, 29]. Such genes include *Fosl1, Pou2f1, Ets1*, as well as *Ifngr1* consistent with another study [30]. Finally, Cluster 3 comprised genes up‐regulated upon IFN‐I exposure, independently of the genotype. Pathway enrichment analysis showed enrichment for genes involved in IFN responses and anti‐viral defense (Figure 1B). Multiple known ISG were found in this cluster [24, 31]. ISG have been classified into several categories based on their transcriptional regulation [24]. Genes from all categories were found in Cluster 3, including genes with a core ISRE motif (such as *Isg15*, *Mx1*, *Ifi44*, *Ifit1*, *Ifit2*, *Ifit3*, *Ifitm3*, *Parp10*, *Stat1*, *Usp18*, *Oas1*, *Oas2*, *Isg20*), genes with a core GAS motif (such as *Irf1*, *Socs3*), and genes predicted to be controlled by other regulators (such as *Cxcl10*, *Cd274*, *Jak2*, *Parp11*, *Slamf7*). To validate these findings in an in vivo context, we injected IFN‐I intra‐peritoneally into CD64*AhR KO mice and WT littermates, and analyzed after 24 h the expression by peritoneal macrophages of ISG at the protein level, using flow cytometry (gating strategy shown in Figure S1A). We found that CD317 (encoded by *Bst2*) and Sca‐1 (encoded by *Ly6a*) were induced by IFN‐I exposure at similar levels in AhR‐deficient and WT peritoneal macrophages (Figure 1C).

**FIGURE 1:**
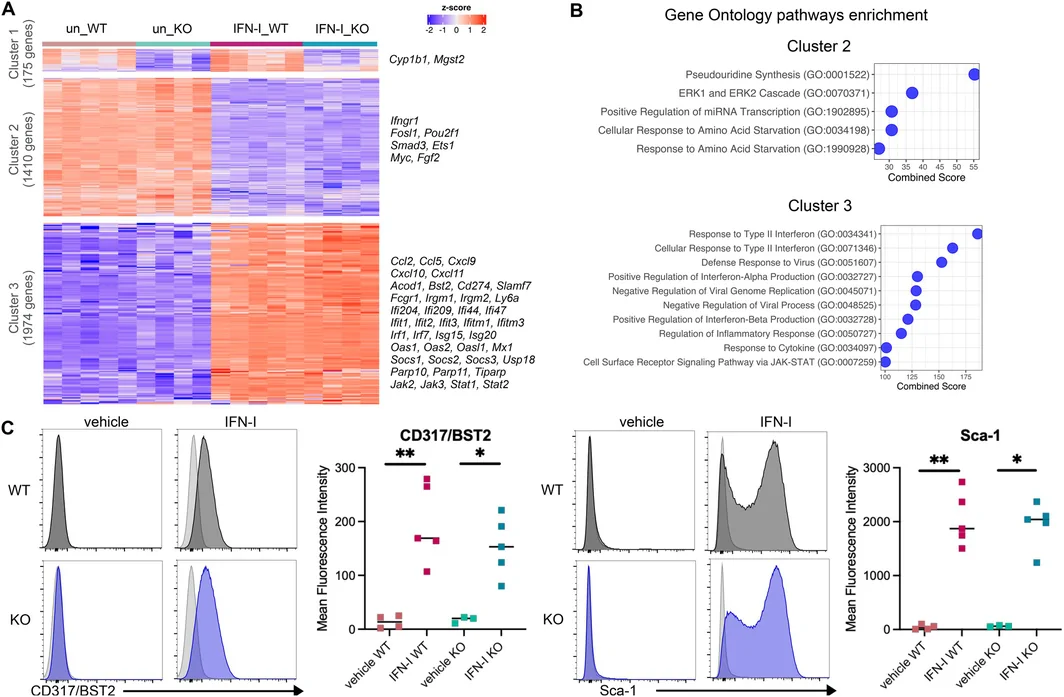
AhR‐deficient and WT macrophages display similar canonical ISG responses. (A, B) Bone marrow derived macrophages from CD64*AhR KO mice and WT littermates were exposed or not to type I interferon (IFN‐I) for 3 h and analyzed by RNA‐seq analysis (*n* = 4–5 biological replicates per group). (A) K‐means clustering (*k* = 3) was performed on differentially expressed genes across conditions (un = unstimulated). Scaled expression of genes from each cluster is shown. Example genes from each cluster are indicated. (B) Pathway enrichment analysis for genes from Clusters 2 and 3 using the Gene Ontology database. (C) CD64*AhR KO mice and WT littermates were injected intra‐peritoneally with type I interferon (IFN‐I) or vehicle control, and peritoneal macrophages were analyzed after 24 h by flow cytometry for CD317/BST2 and Sca‐1 expression. Light gray histograms represent fluorescence‐minus‐one background controls. Median is shown (*n* = 3–4 for vehicle and *n* = 5 for IFN‐I). Kruskal–Wallis test, *p < 0.05, **p < 0.01. Absence of stars indicates “not significant.”

Collectively, these results indicate that constitutive AhR deficiency does not impair the early canonical transcriptional response to IFN‐I in macrophages.

In studies employing lung adenocarcinoma cells, AhR was shown to be required for the baseline expression of molecules of the JAK/STAT pathway, including *Jak2*, *Stat1*, and *Irf1* [16, 17], and for the IFN‐I‐driven upregulation of the regulatory molecule *Cd274* [17]. By contrast, we found that in bone marrow‐derived macrophages AhR deficiency did not affect baseline expression nor IFN‐I‐dependent upregulation of these genes (Figure 2A). To confirm this observation in an in vivo context, we analyzed the expression of CD274/PDL1 by peritoneal macrophages of CD64*AhR KO mice and WT littermates injected with IFN‐I intra‐peritoneally as above. We found that CD274 expression was induced by IFN‐I exposure, and there was no difference between AhR‐deficient and WT peritoneal macrophages (Figure 2B,C). These observations indicate that AhR is dispensable for the IFN‐I‐driven induction of CD274 expression in macrophages.

**FIGURE 2:**
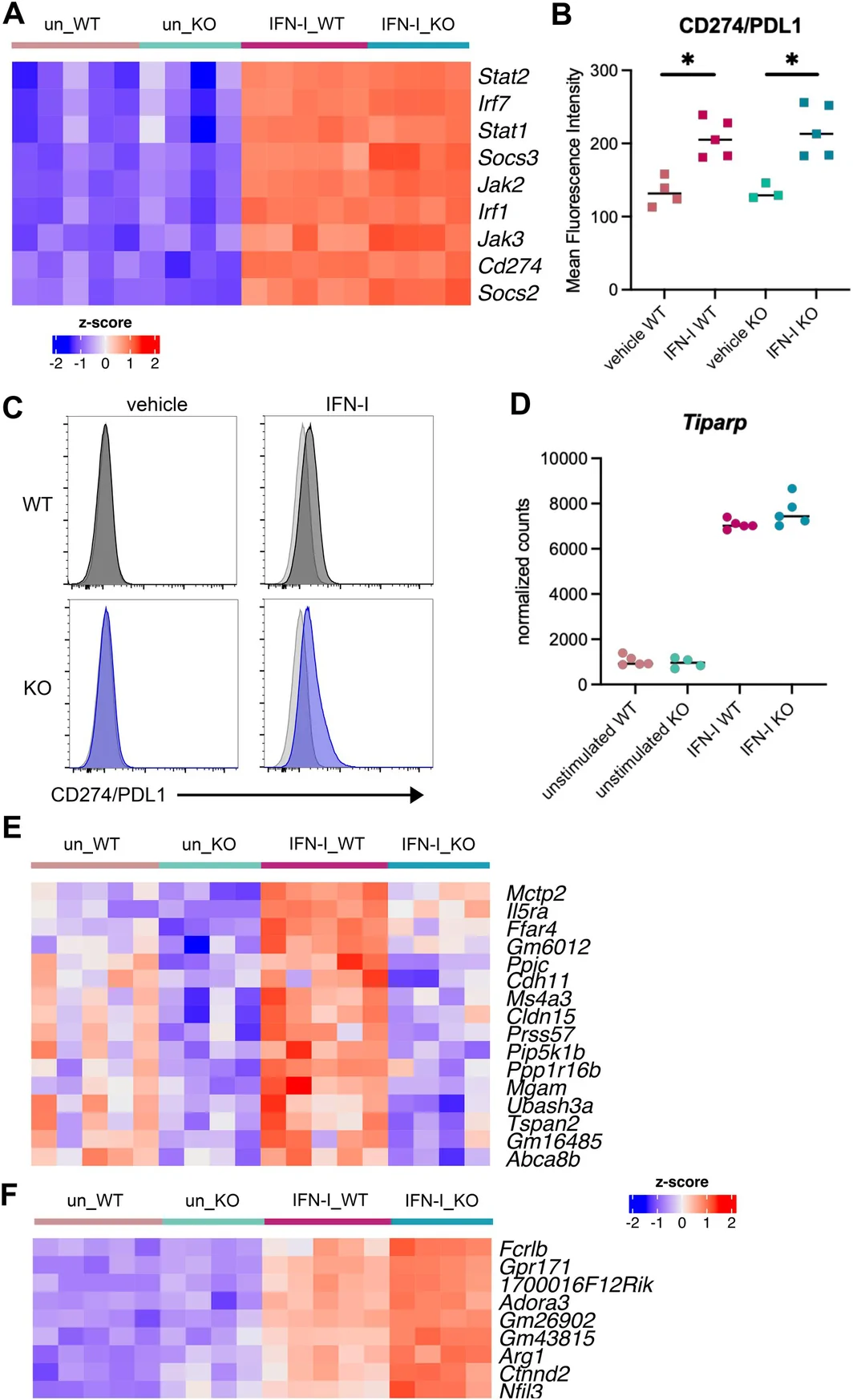
AhR deficiency has minimal impact on transcriptional responses to type I interferon in bone marrow‐derived macrophages. (A, D–F) Bone marrow derived macrophages from CD64*AhR KO mice and WT littermates were exposed or not to type I interferon (IFN‐I) for 3 h and analyzed by RNA‐seq analysis (*n* = 4–5 biological replicates per group). (A) Scaled expression of selected genes (un = unstimulated). (B, C) CD64*AhR KO mice and WT littermates were injected intra‐peritoneally with type I interferon (IFN‐I) or vehicle control, and peritoneal macrophages were analyzed after 24 h by flow cytometry for CD274/PDL1 expression. Light gray histograms represent fluorescence‐minus‐one background controls. Median is shown (*n* = 3–4 for vehicle and *n* = 5 for IFN‐I). Kruskal–Wallis test, *p < 0.05. Absence of stars indicates “not significant.” (D) Normalized counts for *Tiparp*. Median is shown. Each point represents a biological replicate. (E, F) Scaled expression of differentially expressed genes from the interaction analysis “genome/treatment” (un = unstimulated).

Of note, *Tiparp* (also known as *Parp7*), a well‐established AhR target gene involved in its negative feedback loop [32], was identified in Cluster 3 and its expression was unaffected by AhR deficiency (Figure 2D). *Tiparp* expression is induced by several AhR agonists in multiple cell types, and chromatin immunoprecipitation experiments have demonstrated the binding of AhR to response elements upstream of the *Tiparp* promoter region [19, 33, 34]. Tiparp was also reported to be a central player in the negative feedback loop that restrains IFN‐I signaling [19, 35, 36], and was found to be an ISG [35]. Our results show that in bone marrow‐derived macrophages *Tiparp* expression can be induced by Type I interferon through an AhR‐independent mechanism. This suggests the existence of distinct regulatory elements of *Tiparp* expression, downstream of both AhR, and IFNAR signaling. Consistent with this, re‐analysis of public ChIP‐seq data from IFN‐I‐stimulated bone marrow‐derived macrophages showed the binding of STAT1, STAT2, and IRF9 to the promoter region of the *Tiparp* locus (Figure S1B).

Finally, we formally tested the interaction of genotype over treatment. We identified 16 genes for which IFN‐I‐driven upregulation was decreased by AhR deficiency (Figure 2E) and 11 genes for which it was increased (Figure 2F). Statistics for differential expression are reported in Table S2. Pathway enrichment analysis was not performed due to the limited number of genes.

Taken together, our results indicate minimal impact of AhR deficiency on IFNAR‐dependent signaling in bone marrow‐derived macrophages at an early time point.

Our observations contrast with previous reports of AhR interactions with STAT1 signaling [15, 16, 17, 18, 19]. Overall, these findings support the notion of context dependence for the crosstalk between IFNAR and AhR signaling that may include in particular cell type, AhR expression levels, AhR ligand availability, STAT1/STAT2 stoichiometry, and timing.

## 3 Limitations of the Study

A limitation of this study is that AhR was constitutively deleted in macrophages. Compensatory mechanisms during macrophage differentiation could exist and mask an acute AhR contribution to JAK/STAT signaling. Another potential limitation is that only one time point was investigated for in vitro gene expression analysis (3 h post IFN‐I) and for in vivo protein expression analysis (24 h post exposure). We cannot exclude that AhR deficiency impacts macrophage responses to IFN‐I at earlier or later time points. In addition, we restricted our differential analysis to genes showing at least a 2‐fold difference between WT and AhR‐deficient macrophages. We therefore cannot exclude that AhR also modulates more modest changes in expression that may still be biologically relevant. Detecting such changes would require a larger number of biological replicates to achieve sufficient statistical power.

## 4 Material and Methods

### 4.1 Mouse Strains

*Ahr*‐flox (strain 006203) mice were obtained from Jackson Laboratory. *Fcgr1*‐icre were obtained from Bernard Malissen [37]. CD64*AhR strain was generated in‐house by crossing *Fcgr1*‐iCre and *Ahr*‐flox mice. All mice were on C57BL/6J background. Mice were maintained under specific pathogen‐free conditions at the animal facility of Institut Curie in accordance with institutional guidelines. Mice were housed in a 12 h light/12 h dark environment at a temperature of 22°C (±2°C), with *ad libitum* access to water and food. Mice were fed on a standard chow diet (#4RF25SV‐PF1609, aliment pellets, Mucedola). CD64*AhR female mice (AhR^flox/flox^ Cre^+/−^ and Cre^−/−^ littermates) were used at age 8–12 weeks. Efficacy of gene deletion in this strain has been reported before [20]. Animal care and use for this study were performed in accordance with the recommendations of the European Community (2010/63/UE) for the care and use of laboratory animals.

### 4.2 Bone Marrow‐Derived Macrophages Culture

Bone marrow from mouse femur and tibia was flushed out. Cell suspension was cultured with complete RPMI: RPMI‐Glutamax (Gibco) supplemented with penicillin–streptomycin (Gibco) and 10% fetal calf serum (Biosera) in the presence of 80 ng/mL M‐CSF (Miltenyi). After 5 days of culture, cells were exposed to 500 U/mL of Universal Type I IFN Protein (Bio‐techne) for 3 h before lysis for RNA extraction.

### 4.3 RNA‐Seq Library Preparation

For wild‐type macrophages, 5 biological replicates were used. For AhR‐deficient macrophages, 4 biological replicates were used. Cells were lysed in RLT buffer (QIAGEN). Total RNA was extracted using the RNAeasy minikit (Qiagen), including on‐column DNase digestion according to the manufacturer's protocol. The integrity of the RNA was confirmed in BioAnalyzer using the RNA 6000 Nano kit (Agilent Technologies) (8.8 < RIN < 10). Libraries were prepared according to Illumina's instructions accompanying the TruSeq Stranded mRNA Library Prep Kit (Illumina). 500 ng of RNA was used for each sample. Library length profiles were controlled with the LabChip GXTouchHT system (Perkin Elmer). Sequencing was performed using NovaSeq 6000 (Illumina) (100‐nt‐length reads, paired end) with an average depth of 30 million clusters per sample.

### 4.4 RNA‐Seq Data Analysis

Genome assembly was based on the Genome Reference Consortium (mm10). Quality of RNA‐seq data was assessed using FastQC (v0.11.9). Reads were aligned to the transcriptome using STAR [38]. Differential gene expression analysis was performed using DESeq2 (v1.34) with default statistical tests (Wald statistics), using a multi‐factor design (design = ~ genotype + treatment + genotype: treatment) [39]. Genes with low number of counts (sum across all samples < 100) were filtered out. Differentially expressed genes were identified based on adjusted *p*‐value < 0.01 and Log2 FoldChange > 1. Differentially expressed genes were used as input for k‐means clustering of gene expression. The count matrix was normalized using variance stabilizing transformation (VST) then scaled and centered. K‐means clustering was performed with *k* = 3 based on silhouette analysis and elbow methods. The impact of genotype on IFN‐I treatment was assessed by identifying differentially expressed genes using the interaction model. Heatmaps of log2‐scaled expression were generated with *ComplexHeatmap*. The list of genes in each cluster is available in Table S1. Pathway enrichment was analyzed using *EnrichR* [40, 41] and the GO_Biological_Process_2026 database [41, 42]. Enriched pathways were filtered on adjusted *p*‐value < 0.5, odds ratio < 10 and combined score > 20.

### 4.5 ChIP‐Seq Data Analysis

Data from Chromatin immunoprecipitation followed by sequencing (ChIP‐seq) was re‐analyzed from public source (GEO repository, accession number GSE115433). In the original study, ChIP‐seq was performed on mouse bone marrow‐derived macrophages either untreated or after 90 min of exposure to IFN‐ß [43]. Tracks for STAT1, STAT2, and IRF9 binding were visualized at the *Tiparp* locus based on the Genome Reference Consortium (mm10) using Integrative Genome Viewer [44]. H3K4me3 ChIP‐seq track from ENCODE [45, 46] was also added as a marker for the promoter region.

### 4.6 Flow Cytometry

Cells were stained for 30–45 min on ice with indicated antibody cocktails supplemented with Fc block (BD Biosciences) in sterile FACS buffer: PBS (Eurobio scientific) containing 0.5% BSA (Euromedex) and 2 mM EDTA (Invitrogen). After washing with FACS buffer, cells were resuspended in FACS buffer containing DAPI (Fischer Scientific, 100 ng/mL). Cells were acquired on a ZE5 instrument (Bio‐Rad) using volumetric counting. Supervised analysis was performed using FlowJo software v10 (FlowJo LLC).

### 4.7 Peritoneal Cells Analysis

Mice were injected intra‐peritoneally with 5000 U of Universal Type I IFN Protein (Bio‐techne) diluted in 100 μL of PBS. Mice were analyzed after 24 h. Peritoneal lavage was recovered by intraperitoneal injection of 4 mL of sterile PBS.

Cells were stained with anti‐Sca1 BV421 (Biolegend, clone D7), anti‐CD115 APC‐Cy7 (BD Bioscience, clone AFS98), anti‐Ly6G BV510 (Biolegend, clone 1A8), anti‐ICAM2 Alexa488 (Biolegend, clone 3C4), anti‐CD226 PE‐Dazzle594 (Biolegend, clone 10E5), anti‐CD11b Pe‐Cy7 (BD Bioscience, clone M1/70), anti‐CD317/BST2 PE (Biolegend, clone 129C1), and anti‐PDL1 APC (BD Bioscience, clone MIH5).

## Author Contributions

Conceptualization, methodology, and writing: Darawan Tabtim‐On and Elodie Segura. Investigation: Darawan Tabtim‐On. Formal analysis and supervision: Elodie Segura.

## Funding

This work was supported by INSERM, the Agence Nationale de la Recherche (ANR‐10‐LABX‐0043, ANR‐10‐IDEX‐0001‐02 PSL, ANR‐17‐CE15‐0011‐01, ANR‐24‐CE14‐5966), and Fondation pour la Recherche Médicale to Darawan Tabtim‐On (FDT202504020300).

## Conflicts of Interest

The authors declare no conflicts of interest.

## Supporting information


**Figure S1:** (A) Gating strategy for the analysis of peritoneal macrophages by flow cytometry. Representative plots are shown. (B) Public ChIP‐seq data (GSE115433) was analyzed for the binding of STAT1, STAT2, and IRF9 at the *Tiparp* locus in mouse macrophages. H3K4me3 ChIP‐seq from ENCODE is also shown as a marker for the promoter region. ChIP‐seq was performed on bone marrow‐derived macrophages either untreated (baseline) or after 90 min of exposure to IFN‐ß (IFN‐I). Integrative Genomics Viewer tracks are shown.

**Table S1:** List of genes in each cluster of K‐means clustering.

**Table S2:** Differentially expressed genes from the interaction analysis “genome/treatment.”

## Data Availability

Sequencing data is accessible in GEO under accession number GSE315853.
