# Supplementary material for: Aryl Hydrocarbon Receptor Is Dispensable for the Early Transcriptional Response of Mouse Macrophages to Type I Interferon
Source: FASEB J. 2026 Nov 11;40(22):e72429. doi: 10.1096/fj.202604796R (PMC13753966; doi:10.1096/fj.202604796R)
Supplement: Supplementary file 1 [file FSB2-40-e72429-s003.pdf]

Figure S1

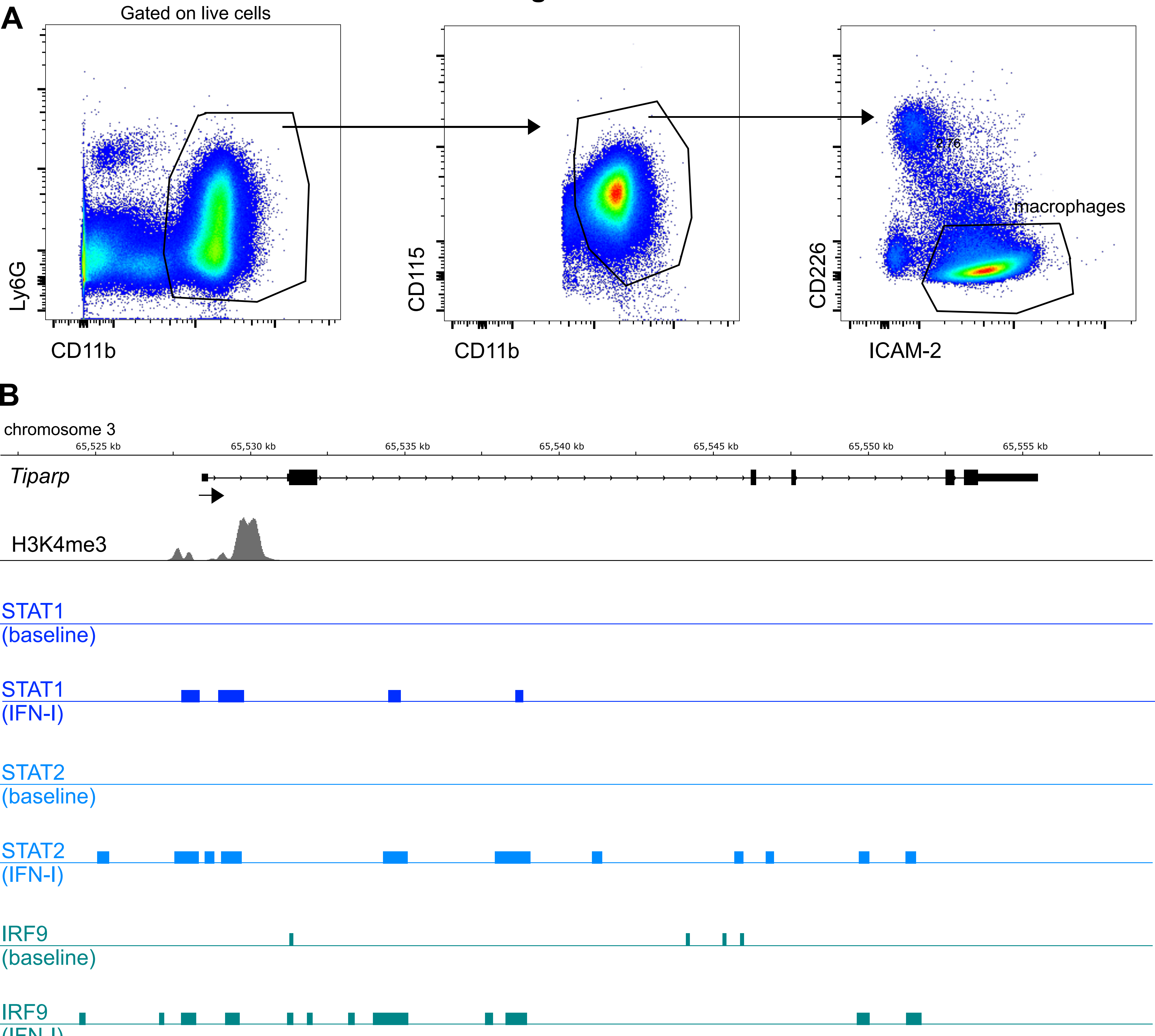
A
Gated on live cells
Ly6G
CD11b
CD115
CD11b
CD226
ICAM-2
2.76
macrophages
B
chromosome 3
65,525 kb
65,530 kb
65,535 kb
65,540 kb
65,545 kb
65,550 kb
65,555 kb
Tiparp
H3K4me3
STAT1 (baseline)
STAT1 (IFN-I)
STAT2 (baseline)
STAT2 (IFN-I)
IRF9 (baseline)
IRF9 (IFN-I)
